# Supplementary material for: Adsorbate-induced lattice deformation in IRMOF-74 series
Source: Nat Commun. 2017 Jan 9;8:13945. doi: 10.1038/ncomms13945 (PMC5228029; doi:10.1038/ncomms13945)
Supplement: Supplementary Information — Supplementary Figures, Supplementary Tables, Supplementary Methods and Supplementary References [file ncomms13945-s1.pdf]

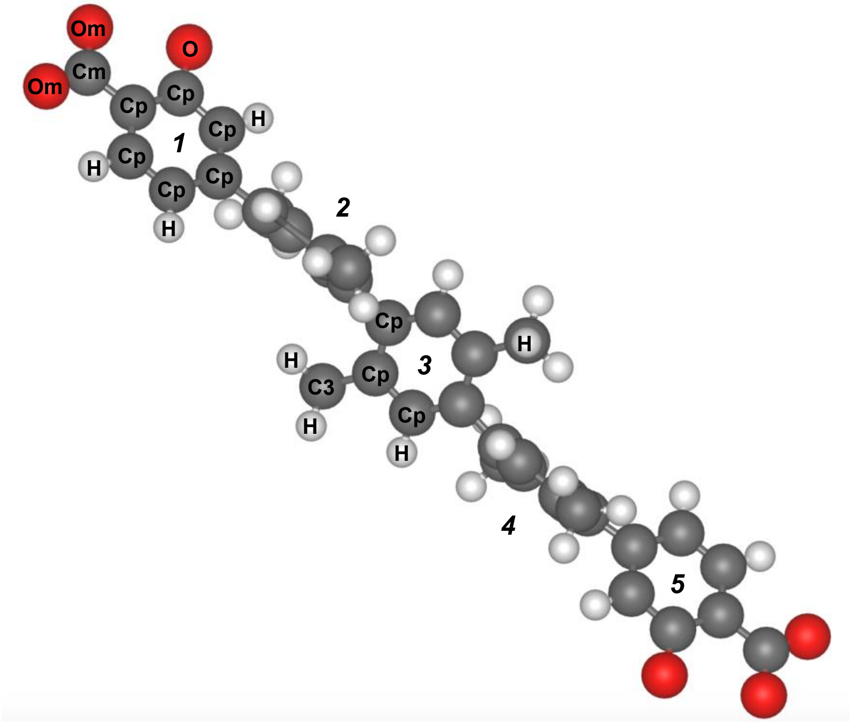

Supplementary Figure 1. Labeled atom types of an IRMOF-74-V linker. The five benzene rings on the linker are numbered. The atoms on benzene *1* are equivalent to the atoms on benzene *5*. Benzenes *2* and *4* (tilted out of the plane of the image) contain the same atoms with the same connectivity as benzene *3*. The equivalent CVFF[1] atom type for each labeled atom type is shown in Supplementary Table 1.

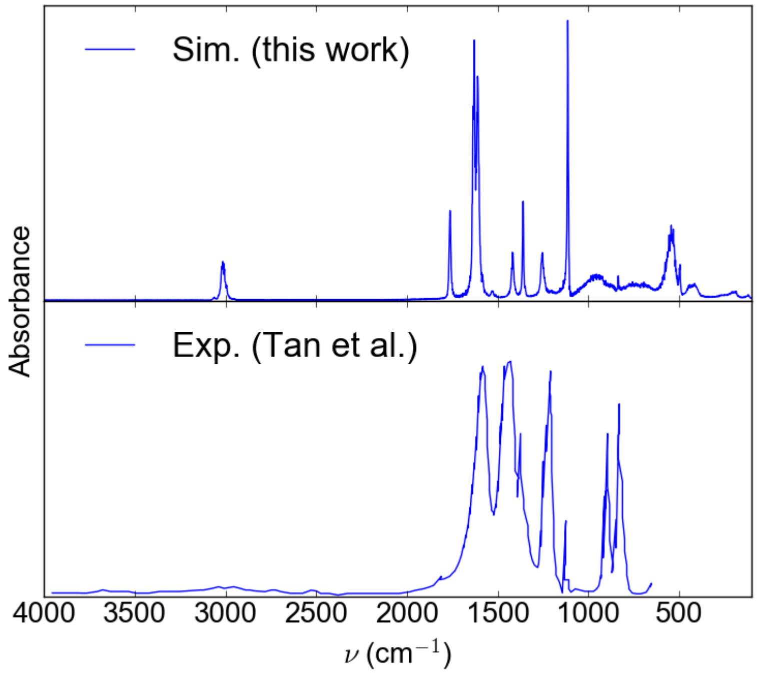

Supplementary Figure 2. Simulated and experimental IR spectra of IRMOF-74 at room temperature. To obtain insight into the reliability of the force field, we have calculated an infrared (IR) spectrum for IRMOF-74 (also known as Mg-MOF-74 or Mg_2(dobdc)) and compared our results to the experimental spectrum measured by Tan *et al*[2]. Where experimental data is provided, there is reasonable agreement between the simulated and experimental spectra. The simulated peaks near 500 cm^{-1} can be attributed to Mg-O bonds[3].

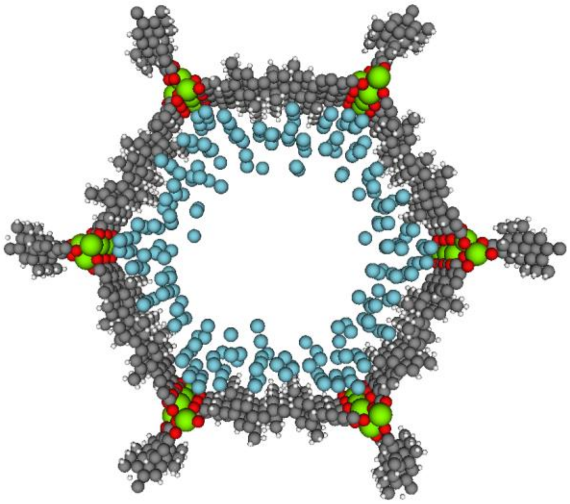

Supplementary Figure 3. Effectively neglecting cross-channel Ar-Ar interactions. To turn off cross-channel Ar-Ar interactions, we simulate adsorption in a single hexagonal channel. We essentially applied periodic boundary conditions for Ar-host interactions with truncated potentials by replicating the crystal structure enough to include all MOF atoms within a distance 10 Å of the boundary of the hexagonal channel. For Ar-Ar interactions, we applied periodic boundary conditions down the channel (the *a*-direction in the CIF file provided), but not in the plane parallel to the page (the *b*-*c* plane in the CIF file provided, *574_charges.cif*).

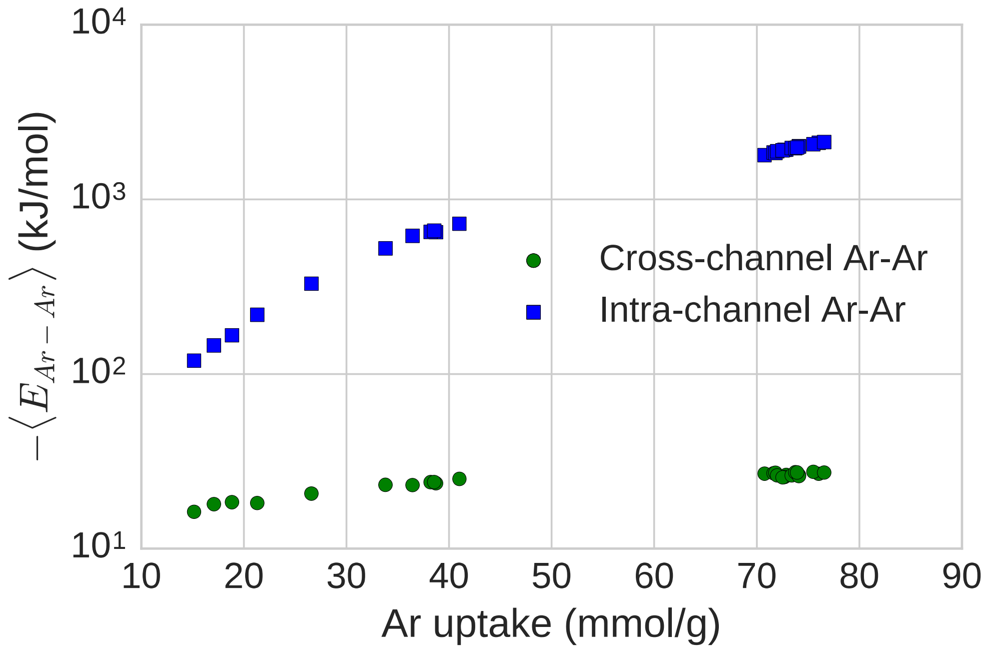

Supplementary Figure 4. Partitioning interactions of Ar in the home channel into cross-channel and intra-channel interactions. Shown is the resulting ensemble average interaction energy of Ar in the channel $<E_{Ar-Ar}>$ during an *NVT* simulation with T = 87 K, partitioned into intra-channel and cross-channel interactions. The *x*-axis shows the *N* (loading) in the *NVT* simulation. Note that the *y*-axis is on a log scale: this shows that the intrachannel interactions are an order of magnitude greater than the cross-channel Ar-Ar interactions.

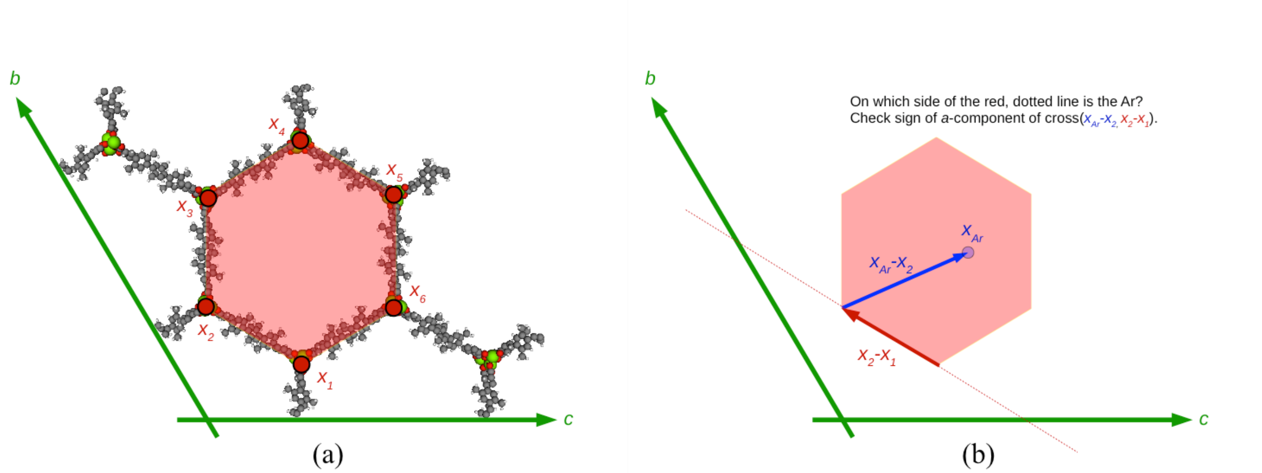

Supplementary Figure 5. Classifying an Ar as belonging or not belonging to the home channel. (a) The home channel is defined by the hexagon in the *b*-*c* plane shown filled in red. The vertices x_i are labeled. (b) Consider our algorithm for $i = 2$. Shown are the two vectors of interest, $x_2 - x_1$ and $x_{Ar} - x_2$ in red and blue, respectively. The line that passes through x_1 and x_2 is shown as the red, dotted line. If the Ar belongs to the home channel, then x_{Ar} lies above this line. To determine whether x_{Ar} is above or below the line, we check the sign of the *a*-component of the cross product of $x_{Ar} - x_2$ and $x_2 - x_1$ after we extend them to three-dimensions by defining the *a*-component to be zero.

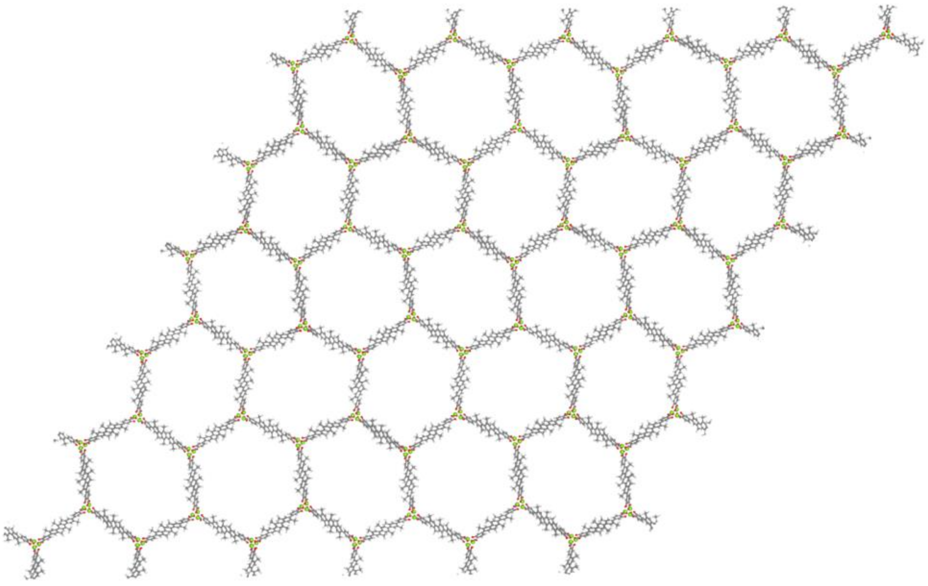

Supplementary Figure 6. Snapshot of a larger (6x6 channel) deformed simulation box. The 6x6 channel simulation box was simulated at the same conditions as the deformed 4x4 channel simulation box (loading commensurate with 0.425 bar).

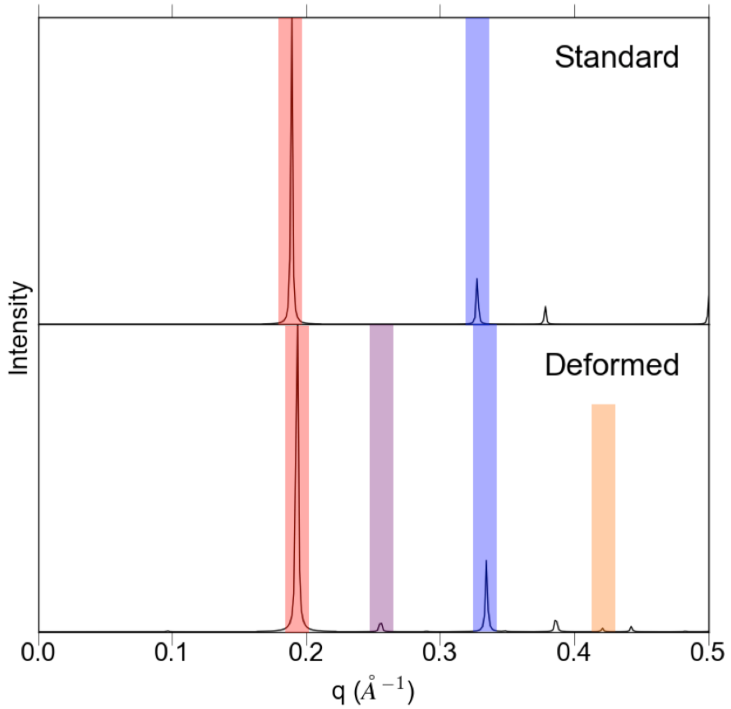

Supplementary Figure 7. Simulated X-ray spectra of standard and deformed (6x6 channel) IRMOF-74-V lattices. A comparison of this deformed x-ray pattern and Figure 6a in the main text shows that the standard (red and blue) peaks shift less in the deformed lattice for the larger simulation box.

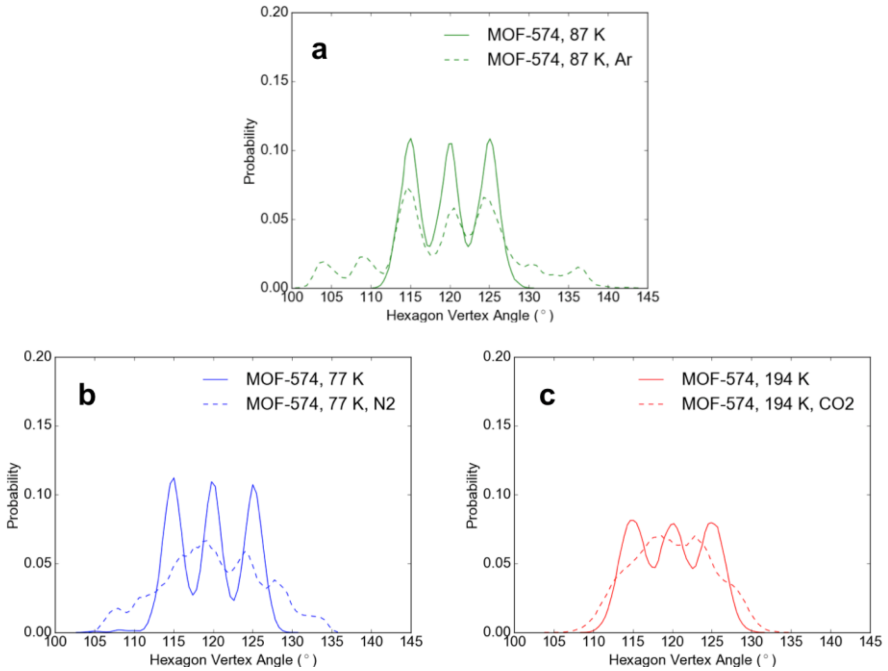

Supplementary Figure 8. Hexagon vertex angle distributions in standard and deformed lattices. The differences between the vertex angle distributions (hexagonal channels) of the lattices deformed by Ar, N_2 and CO_2 and the standard lattices at their respective temperature. The lattices deformed by the more complex adsorbates (N_2 and CO_2) fluctuate more and their peaks are more difficult to distinguish, especially in the case of CO_2. However, a comparison of the range of these three distributions yields information about the extent of deformation. The range of the deformed distributions shows the following order of extent of deformation (from most to least): Ar at 87 K > N_2 at 77 K > CO_2 at 194 K.

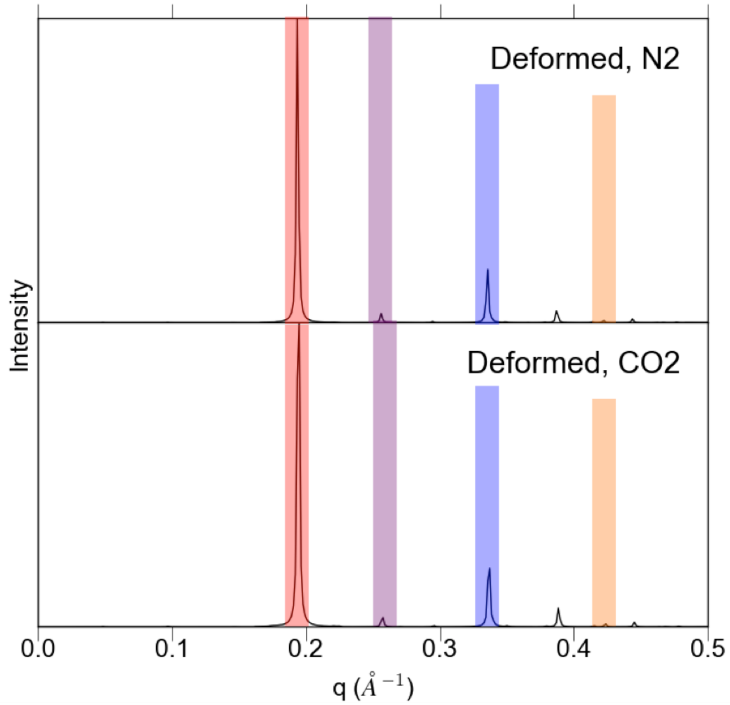

Supplementary Figure 9. Simulated X-ray spectra of IRMOF-74-V lattices deformed by N_2 and CO_2 adsorbates. The N_2 and CO_2 lattices were simulated at 77 K and 194 K, consistent with experimental data. The X-ray pattern of snapshots of the N_2- and CO_2-deformed lattices are similar to the X-ray pattern of the 6x6 channel lattice shown in Supplementary Figure 7, where the deformation is less perfect and leads to the absence of the smallest peak at q = 0.10 A^{-1}.

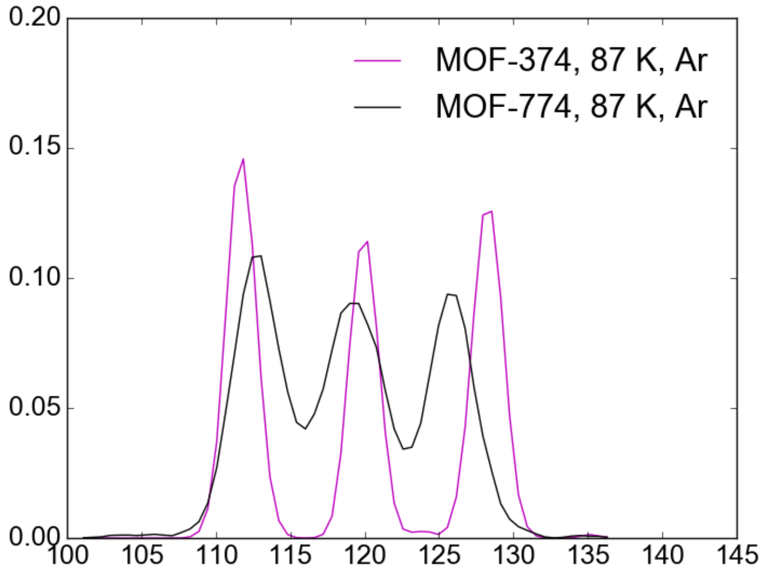

Supplementary Figure 10. Hexagon vertex angle distributions of IRMOF-74-III and IRMOF-74-VII loaded with Ar. The pattern of three peaks resembles the pattern shown for standard MOF-574 in Supplementary Figure 8, indicating that these lattices do not deform even upon adsorption of Ar.

Supplementary Table 1. Equivalence of labeled atom types in Supplementary Figure 1 to CVFF atom types. All information on nonbonded, bond, angle, dihedral and torsional interactions is obtained from the tables in the original CVFF paper[1].

**Labeled Atom Type**	**CVFF[1] Atom Type**	**Description**
Cm	C-	Carboxylic acid carbon
Cp	Cp	Aromatic carbon
C3	C3	Aliphatic carbon
O	O	Alcoholic oxygen
Om	O-	Carboxylic acid oxygen
H	H	Hydrogen

Supplementary Table 2. IRMOF-74-V unit cell parameters predicted by DFT and the force field. The force field unit cell parameters are obtained by allowing the simulation box to deform during energy minimization.

**Parameter (*p3* plane group)**	DFT	Force Field	% difference from DFT
a (Å)	6.8185	6.7737	0.657 %
b (Å)	38.4122	38.2597	0.397 %
c (Å)	38.4122	38.2597	0.397 %
α (°)	119.653	119.640	0.011 %
β (°)	93.3921	93.3799	0.003 %
γ (°)	93.3921	93.3799	0.029 %

Supplementary Methods

In the metal force field developed by Duarte *et al*., the charge distribution on the central metal bead and six dummy beads sums up to a full 2+ charge on the metal ion[4]. As we determined by using the REPEAT charge equilibration scheme, the magnesium atoms in IRMOF-74-V do not have full 2+ charges. Therefore, to use the force field by Duarte *et al*. we scaled the charges on both the central metal bead and the six dummy beads accordingly so that the total charge on the metal complex would be equal to the REPEAT charge. REPEAT charges are made available in the CIF file for IRMOF-74-V, *574_charges.cif*.

Supplementary References

1. P. Dauber-Osguthorpe, V. A. Roberts, D. J. Osguthorpe, J. Wolff, M. Genest, and A. T. Hagler. Structure and energetics of ligand binding to proteins: Escheria coli dihydrofolate reductase-trimethoprim, a drug-receptor system. *PROTEINS*, 4:31-47, 1988.

2. K. Tan, S. Zuluaga, Q. Gong, P. Canepa, H. Wang, J. Li, Y. J. Chabal and T. Thonhauser. Water reaction mechanism in Metal Organic Frameworks with Coordinatively Unsaturated Metal Ions: MOF-74. *Chem. Mater*. 23:6886-6895, 2014.

3. J. T. Luxon, D. J. Montgomery, and R. Summitt. Effect of particle size and shape on the infrared absorption of magnesium oxide powders. *Phys. Rev*. 188:1345, 1969.

4. F. Duarte, P. Bauer, A. Barrozo, B. A. Amrein, M. Purg, J. Aqvist, and S. C. L. Kamerlin. Force field independent metal parameters using a nonbonded dummy model. *J. Phys. Chem. B.*, 118:4351-4362, 2014.
